# Locally advanced rectal cancer patients with mismatch repair protein deficiency can obtain better pathological response after regional chemoembolization

**DOI:** 10.3389/fonc.2023.1131690

**Published:** 2023-04-27

**Authors:** Yuchen Gao, Hualiang Xiao, Wenjun Meng, Juan Liao, Qi Chen, Guowei Zhao, Chunxue Li, Lian Bai

**Affiliations:** ^1^ Department of Gastrointestinal Surgery, Yongchuan Hospital, Chongqing Medical University, Chongqing, China; ^2^ Department of Pathology, Daping Hospital, Army Medical University, Chongqing, China; ^3^ Department of Biotherapy, Cancer Center, West China Hospital, Sichuan University, Chengdu, China; ^4^ Department of General Surgery, Daping Hospital, Army Medical University, Chongqing, China

**Keywords:** locally advanced rectal cancer, neoadjuvant therapy, transcatheter rectal arterial chemoembolization, mismatch repair, pathological complete response

## Abstract

**Background and objective:**

Preoperative transcatheter rectal arterial chemoembolization (TRACE) can enhance the pathological response rate in some patients with locally advanced rectal cancer (LARC). However, how to accurately identify patients who can benefit from this neoadjuvant modality therapy remains to be further studied. Deficient mismatch repair (dMMR) protein plays a crucial role in maintaining genome stability. A proportion of patients with rectal cancer are caused by the loss of mismatch repair (MMR) protein. Given the role of MMR in guiding the efficacy in patients with colorectal carcinoma (CRC), this study is designed to evaluate the effect of dMMR status on the response to neoadjuvant therapy through a retrospective analysis.

**Methods:**

We launched a retrospective study. First, we selected patients with LARC from the database, and these patients had received preoperative TRACE combined with concurrent chemoradiotherapy. Then, the tumor tissue biopsied by colonoscopy before intervention was taken for immunohistochemistry. According to the expression of MLH-1, MSH-2, MSH-6 and PMS-2, these patients were divided into dMMR protein group and proficient MMR (pMMR) protein group. All patients underwent pathological examination at the end of neoadjuvant therapy, either surgically excised tissue or colonoscopically biopsied tissue. The end point was the pathologic complete response (pCR) after TRACE combined with concurrent chemoradiotherapy.

**Results:**

From January 2013 to January 2021, a total of 82 patients with LARC received preoperative TRACE combined with concurrent chemoradiotherapy, and the treatment was well tolerated. Among 82 patients, there were 42 patients in the pMMR group and 40 patients in the dMMR group. 69 patients returned to the hospital for radical resection. In 8 patients, the colonoscopy showed good tumor regression grade after 4 weeks of interventional therapy and refused surgery. The remaining five patients were neither surgically treated nor reexamined by colonoscopy. 77 patients were eventually enrolled in the study. Individually, the pCR rates of these two groups (10%, 4/40 *vs*. 43%, 16/37) showed significant difference (*P* < 0.05). Biomarker analysis indicated that patients with dMMR protein had a better propensity for pCR.

**Conclusion:**

In patients with LARC, preoperative TRACE combined with concurrent chemoradiotherapy showed good pCR rates, especially in patients with dMMR. Patients with MMR protein defects have a better propensity for pCR.

## 1 Introduction

Colorectal cancer (CRC), as the most common type of gastrointestinal malignant tumors, ranks third in incidence and second in mortality globally (1). In the past thirty years, the incidence and mortality of CRC in China have been increasing year by year, and currently account for 18.6% and 20.1% of the cases worldwide, respectively (2, 3). According to statistics, rectal cancer comprises approximately 30% of all CRCs. Rectal cancer is predominately a disease of older individuals, with a marked increase in incidence between 40 and 50 years of age, with increasing risk as a function of advancing age (4). Because the early symptoms of CRC are not obvious, many patients at the time of diagnosis are in the locally advanced stage. Due to the complex anatomy of the rectum and the high local recurrence rate, the treatment and anal preservation in locally advanced rectal cancer (LARC) patients is facing considerable challenges. Currently, the standard treatment for LARC patients is a combination of preoperative neoadjuvant chemoradiotherapy, total mesorectal excision (TME), and postoperative adjuvant chemotherapy (5). Preoperative treatment has been developed to such an extent that the pathological complete response (pCR) rate has reached 25% at the time of surgery (5). However, it is obviously correlated with the occurrence of adverse events, including intestinal dysfunction, leukopenia, sexual dysfunction etc. (6, 7). In recent years, a new treatment combination has emerged for patients with LARC, that is, preoperative transcatheter rectal arterial chemoembolization (TRACE) combined with concurrent chemoradiotherapy followed by radical surgery. Clinical studies have confirmed that this method can significantly improve the pathological response rate of LARC, and the pCR rate can reach about 30% (8). pCR, as an efficient indicator to neoadjuvant therapy, is also an independent predictor of disease-free survival (DFS) and overall survival (OS) in patients with LARC (9), may provide these patients with an alternative to some non-surgical treatments.

According to the recent report, the link between the mismatch repair (MMR) genes and cancer has attracted much attention (10). MMR is an evolutionary conserved mechanism that corrects mutations during DNA replication and damage and plays a critical role in maintaining genomic stability (11, 12). When the MMR gene is mutated, the expression of MMR protein is decreased, not expressed or truncated. Meanwhile the deletion and insertion occurring in the process of DNA replication cannot be corrected, resulting in the instability of genomic DNA. There are as many as 12 MMR genes in humans, among which the four most important genes are MLH1, PMS2, MSH2 and MSH6. These four proteins are the main members of the MMR family, accounting for more than 90% of mutations. The loss of MMR protein expression can lead to the accumulation of mismatch during DNA replication, resulting in the occurrence of microsatellite instability high (MSI-H). About 15% of CRCs are caused by MSI pathway (13). It has been reported that some patients with metastatic CRC are closely associated with MMR protein loss (14, 15). Presently, the significance of deficient MMR (dMMR) protein in the risk assessment, diagnosis and prognosis of CRC has been reported. It is associated with 85% of hereditary non-polyposis CRC (Lynch syndrome) and 15% of sporadic CRC, which has important clinical significance. In 2015, immunotherapy was shown to provide significant clinical benefit in metastatic CRC patients with dMMR/MSI-H status, whereas no response was observed in patients with proficient mismatch repair (pMMR)/microsatellite stability (MSS) status (16–18).

Based on the significance of MMR protein, we hypothesized that preoperative TRACE would be more effective in LARC patients with MMR protein deficiency. To test this hypothesis, we designed and conducted this study. It evaluated the efficacy and safety of preoperative TRACE combined with concurrent chemoradiotherapy in LARC patients with pMMR/dMMR genes. By immunohistochemical method to detect the integrity of the MMR protein in tumor tissues, we can predict the effect of tumor regression according to the lack of MMR protein. This maybe provide a new guiding therapeutic method for patients with LARC whether to continue surgical treatment after neoadjuvant therapy.

## 2 Materials and methods

### 2.1 Participants

Our research was a retrospective and single-center study and approved by the Ethics Committee of Daping Hospital.

The inclusion criteria were as follows: (I) aged 18-80 years old; (II) first and pathological diagnosis with rectal adenocarcinoma of stage T_3-4_N_0_M_0_ or T_1-4_N_1-2_M_0_; (III) the distance between the lower margin of the tumor and the anal margin was ≤12cm; (IV) Eastern Cooperative Oncology Group (ECOG) score of performance status ≤ 1; (V) TRACE combined with concurrent chemoradiotherapy was performed; (VI) the pathological reaction was confirmed by surgical treatment or colonoscopy 4 weeks after the end of radiotherapy; and (VII) no serious heart, lung, liver or kidney dysfunction or immune deficiency disease. The exclusion criteria were as follows: (I) Preoperative examination showed distant metastasis; (II) with other serious complications cannot complete treatment regimen, such as a severe cardiopulmonary dysfunction or a surgical contraindication; (III) had a history of abdominal surgery; (IV) patients with a history of radiotherapy or chemotherapy; and (V) the treatment was not completed or pathological response was not achieved after neoadjuvant therapy.

### 2.2 Study design

Based on the inclusion and exclusion criteria, a total of 82 patients with LARC in the Daping Hospital, Army Medical University from January 2013 to January 2021 were selected. Among them, five patients did not complete the surgical treatment as planned and did not review the colonoscopy after TRACE. Finally, we screened 77 patients for enrollment in the study.

**Figure 1:**
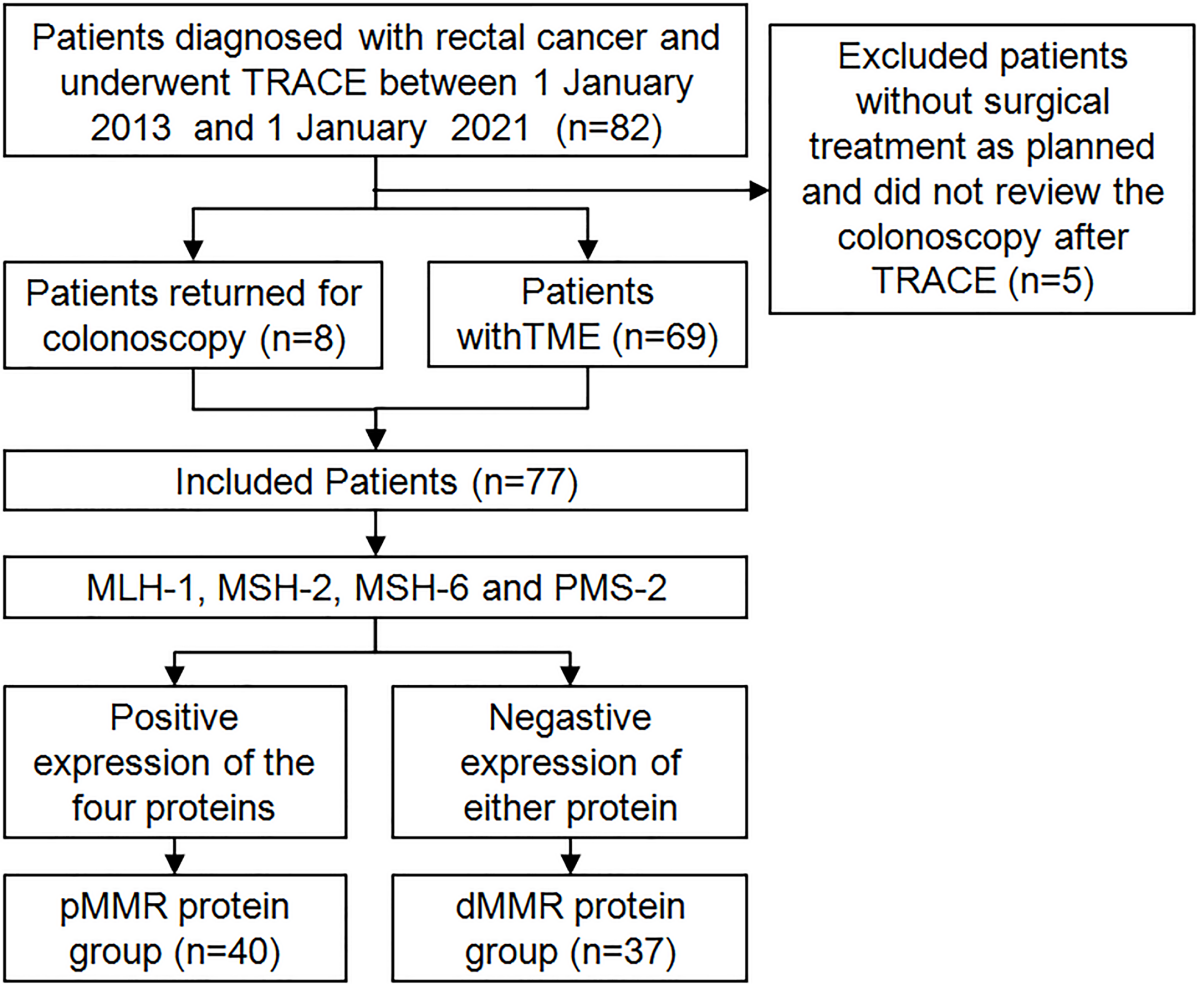
The patients’ recruitment and study design.

All 77 patients underwent immunohistochemistry. Tumor tissue samples were fixed with 10% buffered formalin and embedded in paraffin to prepare 4-micron tissue sections. Immunohistochemical staining was performed by hand using the two-step EnVision (Agilent, Santa Clara, USA) method according to the manufacturer’s instructions. Finally, the immunocomplexes were stained with DAB and observed under a microscope (Olympus, Japan). MLH-1 (ZM-0154), MSH-2 (ZA-0622), MSH-6 (ZA-0541), PMS-2 (ZA-0542), two-step detection kit and DAB color development kit were purchased from Beijing Zhongshan Jinqiao Biotechnology Co., Ltd. The results of immunohistochemical staining were interpreted according to the literature (19). The positive criteria for MLH-1, MSH-2, MSH-6, and PMS-2 were tumor cell nuclei staining and positive nuclei of normal intestinal mucosa, tumor stromal cells, and inflammatory cells in each section. If normal intestinal mucosa, interstitial cells and inflammatory nuclei were positive but cancer cells were negative, the protein expression was absent. Loss of expression of either protein was defined as dMMR, and positive expression of the above four proteins is pMMR. All stained sections were evaluated by two attending physicians of the pathology department of our hospital. If the results of the two physicians were different, the results were re-evaluated. According to the results of immunohistochemical staining of MLH-1, MSH-2, MSH-6 and PMS-2, the patients were divided into pMMR group (n=40) and dMMR group (n=37). **Figure 1** shows the patients’ recruitment and study design.

### 2.3 Treatment regimen

**Figure 2:**
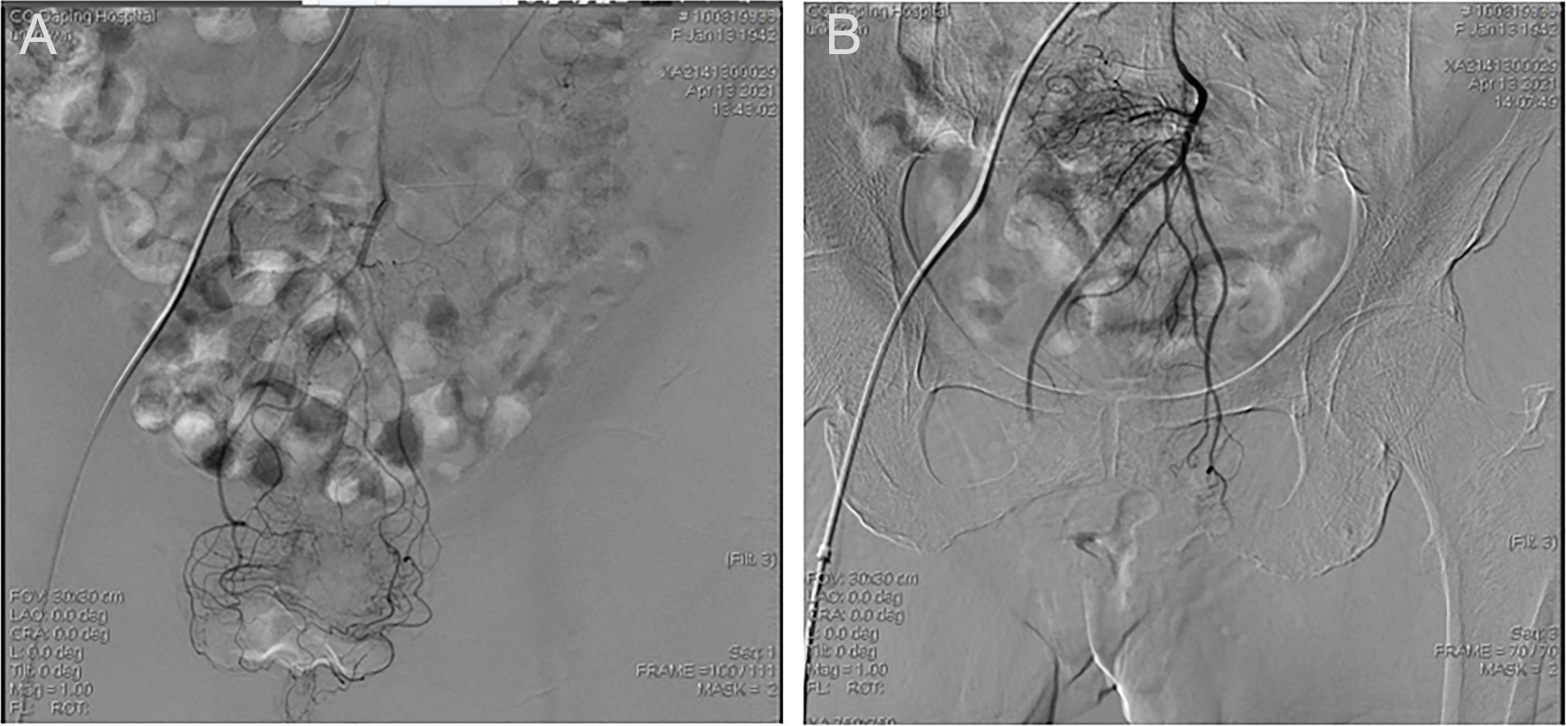
Representative images of a LARC patient undergoing TRACE. **(A)** Frontal inferior mesenteric artery iodixanol angiography. **(B)** Frontal superior rectal arteriogram after TRACE.

All LARC patients underwent femoral artery puncture and digital subtraction angiography. Super-selective catheterization of the superior and inferior rectal arteries was performed using a guidewire. Iodixanol angiography was performed in order to determine the primary artery supplying the tumor. Oxaliplatin (Jiangsu Hengrui Pharmaceutical Co., Ltd., Nanjing, China) was calculated based on body surface area (BSA), diluted into 50 mL of normal saline, and slowly injected through a catheter. Gelatin sponge granules (350-560 μm; Alicon Hangzhou, China) and a 15mL iodixanol injection were embolized into the superior rectal artery. The ultimate embolization results were confirmed by angiography. **Figure 2** showed the representative images of a LARC patient undergoing TRACE. All LARC patients started oral S-1 (Taiho Pharmaceutical Company, Tokyo, Japan) after TRACE. The dosage of S-1 was calculated according to BSA (BSA < 1.2 m^2^, 40 mg, 2x/day; BSA from 1.2 m^2^ to 1.5 m^2^, 50 mg, 2x/day; BSA > 1.5 m^2^, 60 mg, 2x/day). S-1 treatments were administered from day 1 to day 28, and then stopped for 14 days. All LARC patients received 5 weeks of radiotherapy concurrently with the S-1 treatments. The radiation dosage was 1.8 Gy/day, 5x/week, for 5 weeks for a total dose of 45 Gy. The irradiation sites included primary lesions and pelvic lymph nodes.

Radical rectal cancer resection was divided into abdominal perineotomy and low anterior resection and performed 4 weeks after the end of chemoradiotherapy. The specific surgical site depended on a preoperative imaging examination and an intraoperative exploration. Sigmoidostomy or ileostomy was routinely performed, and stoma closure was performed 4-6 months after surgery. mFOLFOX6 (oxaliplatin, leucovorin, and fluorouracil) or CapeOx (capecitabine and oxaliplatin) regimen was recommended for 4 to 6 months as the adjuvant chemotherapy. mFOLFOX6 regimen consisted of oxaliplatin at a dose of 85mg/m^2^, leucovorin at a dose of 400mg/m^2^ and fluorouracil at a dose of 400 mg/m^2^ on day 1. The next day fluorouracil was continued given intravenously. CapeOx regimen consisted of intravenous infusion of oxaliplatin at a dose of 130mg/m^2^ on day 1, followed by oral administration of capecitabine 1000mg/m^2^ from day 1 to day 14, with a 7-day suspension and a 3-week course.

### 2.4 Evaluation of efficacy and safety

Resected surgical specimens were classified according to the eighth edition of the American Joint Committee on Cancer (AJCC) tumor-node-metastasis (TNM) system (20). All resected specimens were continuously sampled and transected during sectioning, and the sections were stained with Hematoxylin-Eosin staining. All lymph nodes were examined according to standard procedures. All sections were independently evaluated by two experienced pathologists. The absence of residual carcinoma in the resected primary tumor tissue and lymph nodes in all sampling areas was defined as pCR. Tumor regression grade (TRG) was assessed according to the 7th edition of the American Joint Committee on Cancer (AJCC) standard (21). TRG0 represented a complete tumor response to the treatment and the absence of tumor cell residue; TRG1 represented a moderate tumor response to the treatment, with a single or small cluster of cancer cells remaining; TRG2 represented a mild tumor response to the treatment with residual tumor cells; and TRG3 indicated poor tumor response to the treatment with few or no tumor cell regression.

During the treatment, the LARC patients’ physical condition was assessed weekly, including vital sign measurements and general medical examinations. Treatment toxicity was graded according to the Common Terminology Criteria for Adverse Events (CTCAE; version 5.0) issued by the National Cancer Institute (22).

### 2.5 Study endpoints and statistical analysis

The main observation indicator was the proportion of LARC patients who achieved pCR. Secondary endpoints included the incidence of adverse events and surgical complications, R0 resection rate, tumor stage, DFS and OS. Adverse events were recorded and graded from the time patients signed an informed consent until 90 days after surgery.

IBM SPSS statistical software (version 26.0) was used for data processing and statistical analyses. The continuous data of LARC patients with a normal distribution were presented as mean and standard deviation, and an independent sample t-test was performed between the dMMR and pMMR protein groups. If the measurement data did not conform to a normal distribution, median and interquartile (P_25_, P_75_) were used, and Mann-Whitney U test was performed for the dMMR and pMMR protein groups. Categorical data were expressed as ratios (percentages), and the dMMR and pMMR protein groups were compared by a chi square test or Fisher exact test. Kaplan-Meier method was used to evaluate survival analysis including DFS and OS between the two groups. All *P*-values were assessed by two-tailed tests, and the significance threshold was set to α = 0.05. *P* < 0.05 was considered statistically significant.

## 3 Results

### 3.1 Patient characteristics

Patients’ characteristics are detailed in **Table 1**. There were 30 males and 10 females in the pMMR protein group with an average age of 58.2 ± 11.7 years. There were 24 males and 13 females in the dMMR protein group with an average age of 59.7 ± 9.7 years. There were 17 patients in the pMMR protein group and 12 patients in the dMMR protein group whose tumor distance to the anal margin was less than 5 cm. All LARC patients received radiotherapy during treatment. The mean cumulative radiation doses of the two groups were 43.7 Gy and 43.5 Gy, respectively. There were no significant differences in demographic and disease characteristics between the two groups (*P*> 0.05).

**Table 1:** Demographic characteristics of the patients.

	pMMR protein group (n=40)	dMMR protein group (n=37)	*t/z*/*χ^2^*	*P*-value
Age (years)	58.2 ± 11.7	59.7 ± 9.7	0.578	0.565
Male (%)	30 (75.0)	24 (64.8)	0.943	0.332
ECOG performance status 1 (%)	8 (20.0)	6 (16.2)	0.185	0.667
Tumor length (cm)	5.3 ± 2.4	6.4 ± 3.0	1.862	0.067
Tumor distance (%)			0.830	0.362
<5cm	17 (42.5)	12 (32.4)		
≥5cm	23 (57.5)	25 (67.6)		
Tumor T staging (%)			2.586	0.108
cT_3_	35 (87.5)	27 (73.0)		
cT_4_	5 (12.5)	10 (27.0)		
Tumor N staging (%)			7.181	0.028
cN_0_	7 (17.5)	14 (37.8)		
cN_1_	12 (30.0)	14 (37.8)		
cN_2_	21 (52.5)	9 (24.3)		
Clinical disease staging (%)			0.544	0.467
II	7 (17.5)	9 (24.3)		
III	33 (82.5)	28 (75.7)		
Sphincter preservation (%)	34 (85.0)	27 (73.0)	1.689	0.194
Radiation dose, median (P_25_, P_75_)	45 (40, 45)	44 (39.6, 45)	1.214	0.225
Adjuvant chemotherapy mFOLFOX6	34	29	0.567	0.452
CapeOx	6	8		

### 3.2 Surgical efficacy and safety

In the data analysis, a total of 69 patients underwent radical surgery after preoperative TRACE combined with concurrent chemoradiotherapy. Radical resection was performed in all 40 LARC patients in the pMMR protein group, including 12 patients undergoing transabdominal perineotomy, and 28 patients undergoing low anterior rectum resection. Conventional sigmoidostomy was performed in 39 of the patients. All 40 patients underwent R0 resection and exhibited negative perienteral margins. In the dMMR protein group, transabdominal perineotomy was performed in 4 patients, and low anterior rectum resection was performed in 25 patients. Conventional sigmoidostomy was performed in 26 of the patients. All 29 patients who underwent surgery reached R0 resection and exhibited negative perienteral margins. There were 8 patients in the dMMR protein group who underwent colonoscopy after TRACE and chemoradiotherapy, which showed pCR. Then these 8 patients refused subsequent surgery. Sphincter preservation rates were 85% and 73% of the pMMR and dMMR protein groups, respectively, and there was no statistical difference between the two groups (*P* = 0.194).

The most common adverse events were leukopenia, anemia, and radiation enteritis (**Table 2**). There were no grade 4-5 adverse events during the period. The incidence of adverse events was similar between the dMMR and pMMR protein groups. The incidence of leukopenia was 12.5% (5/40) and anemia was 10.0% (4/40) in the pMMR protein group. The incidence of leukopenia and anemia were both 10.8% (4/37) in the dMMR protein group, respectively. There were 3 cases of radiation enteritis in the pMMR protein group and 4 in the dMMR protein group. All *P*-values were > 0.05, and there were no statistically significant differences in adverse events between the two groups. Anastomotic leakage (5%), incision infection (2.5%), intestinal obstruction (2.5%), and incisional hernia (2.5%) in the pMMR protein group were not significantly different from anastomotic leakage (2.7%), incision infection (2.7%), intestinal obstruction (5.4%), and incisional hernia (0%) the dMMR protein group.

**Table 2:** Adverse events between the two groups (%).

	pMMR protein group (n=40)	dMMR protein group (n=37)	*P*-value
Leukopenia	5 (12.5)	4 (10.8)	1.000
Anemia	4 (10.0)	4 (10.8)	1.000
Radiation enteritis	3 (7.5)	4 (10.8)	0.705
Anastomotic leakage	2 (5.0)	1 (2.7)	1.000
Incision infection	1 (2.5)	1 (2.7)	1.000
Intestinal obstruction	1 (2.5)	1 (5.4)	0.605
Incisional hernia	1 (2.5)	0 (0.0)	1.000

### 3.3 Pathological response

**Figure 3:**
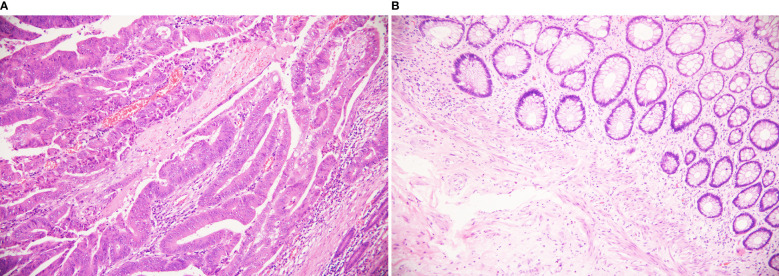
Different pathological findings after TRACE treatment. (HE staining, 200×) **(A)** It shows poor response to tumors, in which still a lot of residual tumor cells. Arrows show tumor cells. **(B)** It shows pCR complete remission without residual tumor cells.

**Figure 4:**
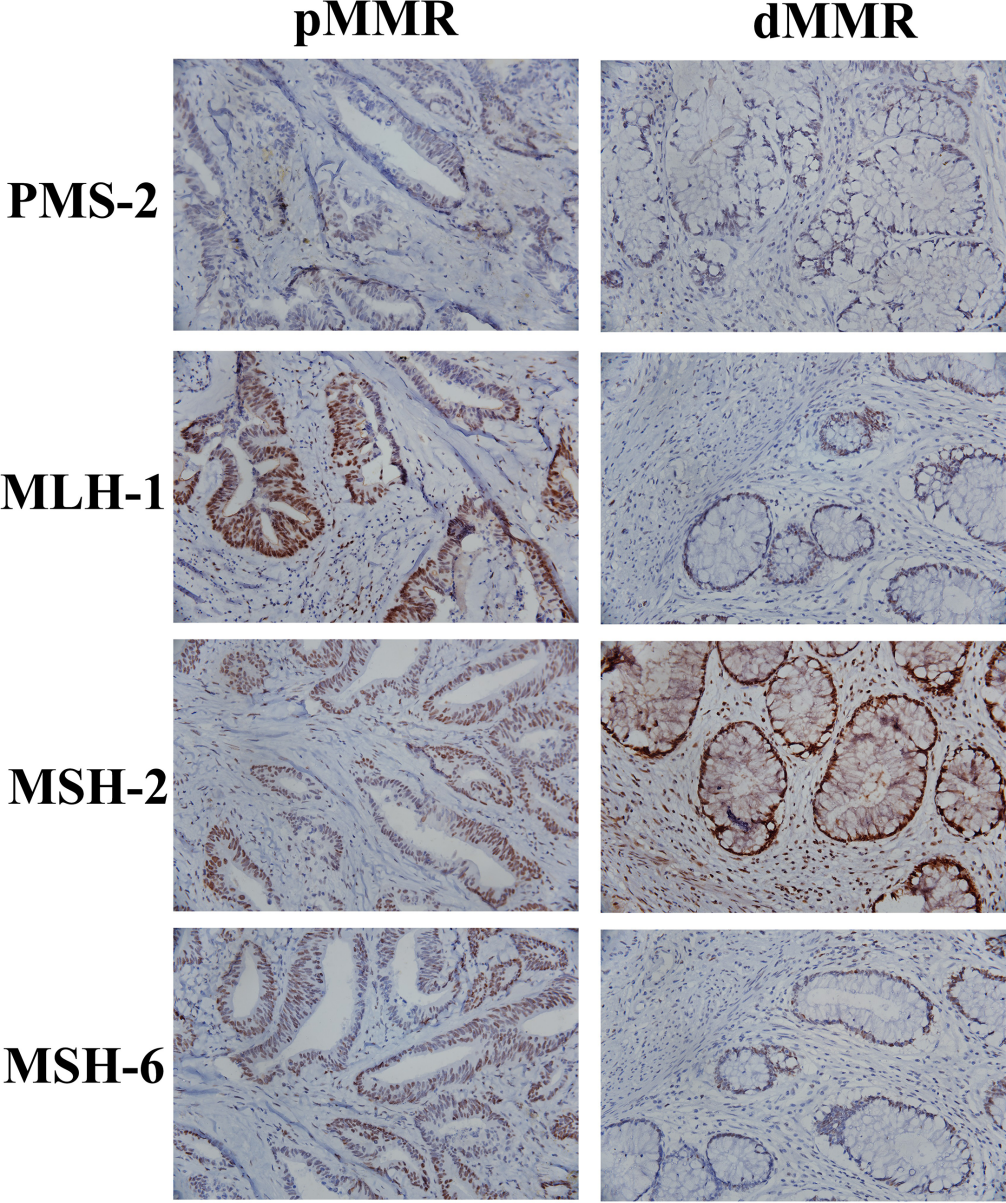
Pathological sections of tumor specimens from patients with pMMR and dMMR (400×).

Preoperative TRACE can lead to pathological reactions in patients with pMMR and dMMR tumors. TRG was assessed according to the AJCC staging scheme, of 77 cases of surgical resection specimens. In the pMMR protein group, there were 4 cases with TRG0, 11 of TRG1, 15 of TRG2, and 10 of TRG3. In the dMMR protein group, there were 16 cases of TRG0, 17 of TRG1, 3 of TRG2, and 1 of TRG3. The positive rate of pCR in all LARC patients after treatment was 26% (20/77). The pCR rate was 10% (4/40) in the pMMR protein group and 43% (16/37) in the dMMR protein group (*P*=0.001). Negative lymph nodes were present in 82% (63/77) of all LARC patients, including 73% (29/40) in the pMMR protein group and 92% (34/37) in the dMMR protein group. **Table 3** showed the comparison of pathological response between the two groups. **Figure 3** shows different pathological findings after TRACE treatment, and one of the dMMR patients’ pathological section from the tumor specimen is shown in **Figure 4**.

**Table 3:** Comparison of pathological response between the two groups (%).

	pMMR protein group (n=40)	dMMR protein group (n=37)	*z/χ^2^*	*P*-value
Pathological response				
pCR (ypT0N0M0)	4 (10.0)	16 (43.2)	11.047	0.001
Non-pCR	36 (90.0)	21 (56.8)		
T category			12.936	0.012
ypT_0_	4 (10.0)	16 (43.2)		
ypT_1_	1 (2.5)	0 (0.0)		
ypT_2_	7 (17.5)	5 (13.5)		
ypT_3_	22 (55.0)	10 (27.0)		
ypT_4_	6 (15.0)	6 (16.2)		
N category			5.561	0.062
ypN_0_	29 (72.5)	34 (91.9)		
ypN_1_	8 (20)	3 (8.1)		
ypN_2_	3 (7.5)	0 (0.0)		
Pathological stage, n (%)			11.922	0.008
0	4 (10.0)	16 (43.2)		
I	8 (20.0)	5 (13.5)		
II	17 (42.5)	12 (32.4)		
III	11 (27.5)	4 (10.8)		
Tumor regression grade			23.769	< 0.001
0	4 (10.0)	16 (43.2)		
1	11 (27.5)	17 (45.9)		
2	15 (37.5)	3 (8.1)		
3	10 (25.0)	1 (2.7)		

### 3.4 Survival data analysis

**Figure 5:**
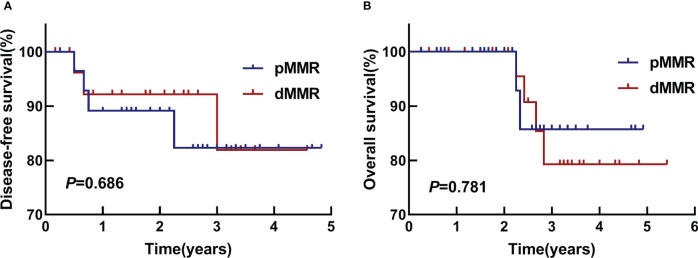
DFS and OS. **(A)** Kaplan-Meier survival analyses of DFS in two groups, *P*=0.686. **(B)** Kaplan-Meier survival analyses of OS in two groups, *P*=0.781.

All survival analyses were reviewed at the last follow-up. The analysis was performed using the available observations of all participants in the study. The follow-up time for both groups was 5 years. There was no statistical difference between the two groups (**Figure 5**). The DFS rates of the two groups in 1 year and 3 years were 89% *vs*. 92%, and 82% *vs*. 82%, respectively (*P*=0.686). The OS rates of them in 3 years were 79.2% *vs*. 85.7%, *P*=0.781.

## 4 Discussion

Our study is the first to propose that MMR can predict pathological responses. In this study involving MMR proteins, patients with LARC were treated with neoadjuvant TRACE combined with concurrent chemoradiotherapy. Only four weeks after treatment, biopsies were taken by endoscopy or pathological examination of surgically resected tissue. Some patients can achieve pCR, while the pCR rate of dMMR patients and pMMR patients is 43.2% and 10%, respectively. Although there was no difference in DFS and OS between the two groups in our study, significant differences could be seen in pathological response. So, we put forward a new experimental study. After the neoadjuvant treatment of preoperative TRACE combined with concurrent chemoradiotherapy in patients with LARC, the MMR protein expression status can be used to guide whether it is necessary to continue surgical treatment. That is, under the guidance of MMR proteins, the pCR rate of patients may be improved, so that patients can choose to wait for treatment instead of radical resection. Moreover, the study provides preliminary evidence.

In NICHE’s study, pCR was achieved in all CRC patients with dMMR protein, 95% of which were primary cancers (≤ 10% of viable residual tumors in surgical specimens), including 12 (approximately 60%) pCR cases (23). One retrospective analysis has demonstrated that high rates of pCR can be achieved with checkpoint inhibition in pretreated dMMR metastatic CRC patients (24). In our study, although both groups received the same neoadjuvant therapy, rectal cancer patients in the dMMR group produced a higher pCR rate and were well tolerated without safety problems. Radiotherapy and surgery may have long-term effects on the bowel and bladder function and immune function of patients (6, 7, 25, 26). These adverse events have a significant impact on the daily life of patients, so if the MMR protein loss can be used to guide the need for surgery in patients with LARC, it may bring greater benefits to this population.

Studies have shown that MMR genes correlate with clinicopathological features of LARC and can be biomarkers that contribute to the prognosis of patients. Meanwhile, MMR gene is an important part of the DNA damage response pathway, which maintains the integrity of the genome and reduces primary mutations. It is mainly composed of a series of repair proteins that specifically repair DNA mismatches, including MLH1, PMS2, MSH2, MSH6, etc. (27). Inactivation of any of these genes can lead to the loss of function of MMR proteins, which may be related to the occurrence and development of endometrial cancer, CRC and ovarian cancer. The relationship between the MMR genotype and pCR rates has increased the interest in options of rectal cancer treatment. As we all know, neoadjuvant immunotherapy has been tested in several solid tumors (28–31), and the level of activity in other tumor types is far less than the degree of activity we have observed in LARC patients with dMMR (32). Therefore, dMMR protein tumors respond better to immunotherapy (33). At the same time, we observed that patients with MMR protein deficiency in our study were more sensitive to TRACE and were prone to achieve pCR.

Neoadjuvant chemoradiotherapy has been a standard treatment for LARC. Traditionally, radical surgery is scheduled 6 to 10 weeks after the end of a long course of chemoradiotherapy. Whereas, the tumor was resected 4 weeks after TRACE. All patients received adjuvant chemotherapy using mFOLFOX6 or CapeOx regimens for 4–6 months. Study shows that regardless MSI, 5-fluoruracile (5-FU) have been the backbone in CRC treatment, being combined with others antitumor drugs such as platinum alkylating agents (34). Therefore, we also chose 5-FU as one of the main anti-tumor drugs after surgery. Studies have shown that both proficient and deficient CSC/MMR showed high 5-FU chemoresistance regardless to MMR status (35). Previous studies have shown that adding neoadjuvant chemoradiotherapy can reduce the tumor stage, reduce distant metastasis, and improve pCR rates in LARC patients; however, there was no difference in DFS or OS compared with the group of adjuvant only (36, 37). Also in our study, there were no statistically significant differences in DFS and OS between the pMMR and dMMR groups. In another study, the overall pCR rate was 25.3% in patients treated with preoperative chemotherapy alone and those treated with both preoperative and postoperative chemotherapy (38). Our study added the MMR proteins on the basis of neoadjuvant therapy. Meanwhile, our research is the first to show that MMR proteins can predict pathological responses. The addition of TRACE before surgery provides better pathological remission rate of more LARC patients versus standard treatment with a similar safety profile (8).In other words, preoperative TRACE provided better pathological remission rate versus standard treatment and did not increase the risk of perioperative and postoperative complications. In particular, preoperative TRACE with concurrent chemoradiotherapy has potential advantages in preventing distant metastasis of LARC. Besides, it also provides an opportunity for the implementation of non-surgical, watch-and-wait strategies to improve quality of life. However, compared with conventional neoadjuvant chemotherapy treatment modalities, TRACE has a higher cost, and patients also have to bear the risk of intestinal necrosis caused by surgery. Based on this study, if the MMR gene can be combined with the treatment mode of TRACE, preoperative TRACE combined with chemoradiotherapy may be more accurate in patients with LARC with pMMR or dMMR and better pathological response.

One question worth considering is why rectal tumors deficient in MMR proteins respond better. Recent evidence suggests that a combination regimen with a single dose of a programmed death 1 blocking agent or cytotoxic T lymphocyte-associated antigen-4 inhibitors is highly effective against advanced MSI-H/dMMR tumors but not against pMMR tumors (16, 39). The exact mechanisms by which MSI-H/dMMR LARC patients develop resistance to immunotherapy are unknown but may be explained by the biodiversity of the host immune system and tumor biology. Drug resistance associated with intra-tumor heterogeneity in MSI-H/dMMR status has been reported (40, 41). In LARC, most hypermutated tumors are MSI-H/dMMR.

MMR proteins may be another potential predictive biomarker. Many preoperative and perioperative treatment strategies have achieved good results in gastrointestinal tumors. Therefore, we propose MMR proteins as a potentially effective tool to guide therapeutic strategies in locally advanced MSI/dMMR and MSS/PMMR CRC populations. Further research should focus on developing novel imaging biomarkers in combination with molecular markers to provide a more accurate assessment, or even prediction, of response to neoadjuvant therapy. Given that our study was an exploratory study with a small sample size, we recommend that the findings be interpreted with caution. Furthermore, patients with dMMR seem more likely to benefit from our neoadjuvant study strategy than patients with pMMR.

Although our results are promising, especially the pCR rate of 42% in the dMMR group, the sample size of this study was small and only represents the results of a single institution. Another significant limitation was that larger-scaled RCTs should have been performed. More importantly, if colonoscopy indicates good pathological regression after neoadjuvant therapy, some patients choose to refuse surgical treatment, and their long-term prognosis cannot be compared. We should add some observation indicators in subsequent studies to follow up the long-term prognostic effect. If confirmed in subsequent large studies, the lack of MMR protein may provide guidance for neoadjuvant therapy in patients with LARC, which can make most patients avoid surgical resection, save the anus, and improve the quality of life. In our preliminary study, TRACE combined with concurrent chemoradiotherapy had a better pathological response rate and short-term efficacy (8, 42). These results lead us to believe positively that in the background of preoperative interventional chemoembolization, disease remission will account for a larger proportion. As the data become more complete and more mature, we envision that MMR proteins will be used in the context of preoperative interventional chemoembolization to evaluate other MMR protein-deficient tumors, such as gastric and prostate cancers. This may open up a new world of neoadjuvant chemotherapy for different types of tumor.

## 5 Conclusion

In conclusion, the prognosis can be predicted according to the absence of MMR proteins such as MLH1, MSH2, MSH6 and PMS2 in clinical treatment of rectal cancer. Then the MMR genotype was used to make the next targeted treatment plan for the patient, such as waiting treatment without surgery. All in all, we found that patients with MMR protein deficiency in our study were more sensitive to TRACE and were prone to achieve pCR. Further prospective studies are needed to verify the efficacy and safety of preoperative interventional chemoembolization in patients with LARC guided by MMR.

## Data availability statement

The original contributions presented in the study are included in the article/supplementary material. Further inquiries can be directed to the corresponding authors.

## Ethics statement

Our research was a retrospective and single-center study, and approved by the Ethics Committee of Daping Hospital.

## Author contributions

YG: writing-original draft preparation, and funding acquisition. HX: immunohistochemistry and reading of pathological sections. JL: data analysis and graphics. QC and GZ: formal analysis and investigation. WM: participation in the discussion, supervision, review, and editing. CL: conceptualization and funding acquisition; LB: conceptualization, funding acquisition, and supervision. All authors read and approved the final manuscript. All authors contributed to the article and approved the submitted version.
